# Cell‐Specific Signal Bias Shapes β3‐Adrenoceptor Profibrotic Activation in Cardiac Fibroblasts

**DOI:** 10.1111/jcmm.71416

**Published:** 2026-10-17

**Authors:** Ali H. Eid

**Affiliations:** ^1^ Department of Basic Medical Sciences, College of Medicine, QU Health Qatar University Doha Qatar

Heart failure therapies have traditionally treated the myocardium as a functional whole. In doing so, we risk overlooking the fibroblast [1]. In this issue, Xu and colleagues show that β3‐adrenergic receptor activation promotes a profibrotic phenotype in cardiac fibroblasts through β‐arrestin2‐mediated biased signalling [2]. The authors demonstrate that this pathway activates the JNK/c‐Jun axis to upregulate TGF‐β1, apparently independently of classical Gi protein coupling [2]. This discovery encourages us to rethink the cellular architecture of cardiac neurohormonal regulation. Sympathetic nervous system overdrive remains an important driver of adverse remodelling in failing hearts. We have long focused on β1‐adrenergic receptor blockade as a central therapeutic strategy. The present findings suggest that we must also account for β3‐adrenergic receptor signalling and its distinct cellular consequences [3].

The work of Xu and colleagues challenges the assumption that β3‐adrenegic receptor signalling is uniformly cardioprotective [2]. For decades, pharmacologists have associated this receptor with Gi‐dependent signalling, including pathways linked to nitric oxide synthase and negative inotropic effects [4]. We now see a change; the receptor behaves entirely differently in fibroblasts. By virtue of recruiting β‐arrestin2, the β3‐adrenoreceptor triggers a profibrotic cascade [2, 5]. This cell‐specific signalling bias may help explain apparently conflicting observations surrounding β‐adrenergic receptor biology [5, 6, 7, 8]. Indeed, the timing of this report aligns well with the industry push towards biased ligands. The findings provide a mechanistic rationale for examining how a single receptor can exert different effects in a cell context‐dependent manner. We must, therefore, recognize the fibroblast as an independent pharmacological context [9].

We must, however, confront a significant methodological limitation regarding the experimental model. The authors rely on receptor overexpression to stimulate β3‐adrenoreceptor signalling [2]. This approach may alter receptor abundance, subcellular distribution, receptor stoichiometry and adaptor recruitment [10]. Physiological receptors reside predominantly on the cell surface, while overexpression may produce non‐physiological receptor pools [10]. Thus, increased β‐arrestin2 recruitment and subsequent downstream signalling could partly reflect receptor abundance rather than an intrinsic property of the receptor signalling mechanism. Furthermore, the study does not map G protein‐coupled receptor kinase‐dependent phosphorylation data. β‐arrestin2 recruitment is influenced by receptor phosphorylation, conformation and cellular context [11]. Without mapping relevant phosphorylation sites, one cannot define the precise structural mechanism underlying the noted biased signalling. The observed profibrotic phenotype could therefore reflect altered receptor clustering rather than bona fide biased agonism. Future investigations should address the specific serine and threonine residues responsible for β‐arrestin2 docking, while also considering how tissue‐specific microenvironmental cues could alter receptor function. Only then will we understand the precise molecular switch controlling this profibrotic transition.

The path forward requires moving beyond neonatal mouse fibroblast cultures. We need to validate these findings in adult human cardiac fibroblasts subjected to mechanical stress. The ultimate test will involve constructing fibroblast‐specific β‐arrestin2 knockout mice. Such a model could more definitely separate the β3‐adrenergic signalling in fibroblasts from potentially cardioprotective signalling in other cardiac cell types. Importantly, the study does not yet establish that β3‐adrenergic receptor signalling represents bona fide pharmacological bias. Quantitatively comparing G‐protein and β‐arrestin2 pathways with appropriate reference ligands will help reveal whether the response reflects ligand‐dependent bias, cell‐specific coupling or both. Indeed, these experiments could provide a strong argument to design β3‐adrenergic ligands selective for the Gi pathway. This can be achieved by screening compounds blocking β‐arrestin2 recruitment while preserving Gi coupling. Such a targeted approach could, in principle, provide a path to halt myocardial fibrosis without compromising cardiac contractility. Blunt β‐blockade shall not, however, be regarded as obsolete. It has already been established that β‐blockers are useful in select patients of heart failure with reduced ejection fraction. Accordingly, the present findings point to a more specific pharmacological question: is it possible to dissociate adrenoceptor signalling by receptor subtype, signalling pathway or cell type?

The true *raison d'être* of the piece lies in its translational potential. A β3‐adrenergic biased ligand might, in theory, preserve the cardioprotective signalling in cardiomyocytes while simultaneously limiting the profibrotic β‐arrestin2 signalling in fibroblasts. Whether this can be achieved safely remains unknown. The authors provide a good molecular blueprint for this endeavour, but not yet a basis for clinical testing in heart failure with preserved ejection fraction. The next steps, I argue, are clear: establish endogenous receptor signalling; structurally define the β3‐adrenoceptor/β‐arrestin2 interaction; validate the pathway in adult and human fibroblasts; and determine its in vivo relevance. The concept of a single receptor exerting different distinct effects in different cell types deserves a closer look. The work of Xu and colleagues represents a step in the correct direction.

## Author Contributions

**Ali H. Eid:** conceptualization, writing – original draft, writing – review and editing.

## Funding

The author has nothing to report.

## Conflicts of Interest

The author declares no conflicts of interest.

## Data Availability

Data sharing not applicable to this article as no datasets were generated or analysed during the current study.
